# Validity of temperature, duration, and vessel seal on 24-hour urinary hydration markers

**DOI:** 10.1371/journal.pone.0220724

**Published:** 2019-08-05

**Authors:** William M. Adams, J.D. Adams, Eleni M. Karras, Erin Rysanek

**Affiliations:** 1 Department of Kinesiology, University of North Carolina at Greensboro, Greensboro, NC, United States of America; 2 Division of Endocrinology, Diabetes and Metabolism, Mayo Clinic, Rochester, MN, United States of America; Nottingham Trent University, UNITED KINGDOM

## Abstract

The purpose of this study was to examine the effect of storage temperature, duration, and storage vessel seal on 24 h urinary hydration markers. Twenty-one males (n = 8) and females (n = 13) (mean±SD; age, 24±5 y; body mass, 68.9±24.2 kg; height, 160.2±32.1 cm) without a history of renal disease or currently taking any medications or supplements known to affect the accuracy of urinary hydration markers were enrolled in this study. Participants provided a 24 h urine sample in a clean container with each urine sample being separate into four separate containers, two in each of the following temperatures: 7°C and 22°C. One specimen container at each temperature was either sealed using the manufacturers cap (single sealed) or the manufacturers cap plus laboratory wrapping film (double sealed). Each sample was analyzed after 1, 2, 3, 7 and 10 days. Urine samples were assessed for urine osmolality (U_OSMO_), urine specific gravity (U_SG_) and urine color (U_COL_). U_OSMO_ was stable at 7°C for two days (mean difference [95% CI]; +1 mmol·kg^-1^ [0+3], p>0.05) and three days (+1 mmol·kg^-1^ [0, +3], p>0.05) for single sealed and double sealed containers, respectively. U_SG_ measures were stable for singled sealed and double sealed for up to ten days when stored at 22°C. U_COL_ measures were maintained for up to three days in all storage methods (p>0.05). In conclusion, if immediate analysis is unavailable, such as in the case of field based or longitudinal research, it is recommended that 24 h urine samples are stored in a refrigerated environment and hydration markers (U_OSMO_ and U_COL_) be assessed within 48 h.

## Introduction

Analysis of urine specimens is widely utilized to determine day-to-day hydration status in free living individuals [1–3] as well as for other markers of health [4–8]. Recommendations outlined by the Clinical and Laboratory Standards Institute indicate that urine specimens should be processed within 3 hours of collection with the specimen being stored on ice to maintain the stability of the sample; however, these recommendations lack robust supportive data [9]. Furthermore, it is oftentimes not feasible in some laboratory and field studies to abide by these recommendations, especially in studies that are subjected to longer storage periods prior to analysis of the specimen.

To date, only three studies [10–12] have examined the effects of temperature and/or duration of storage of urine samples on the stability of hydration assessment measures. Bezuidenhout et al., found urine osmolality to be stable (<1 mosmol•kg^-1^ variation) for up to 36 hours at room temperature [12], whereas Adams et al., found urine osmolality to be comparable (mean difference [95%CI], 8 mmol/kg [–1, 17]) to baseline measures for up to 7 days when stored in a 7°C environment [11]. Conversely, Adams et al. [10] found no difference in storage temperature on urine osmolality measures when assessed 48 hours after baseline.

While these studies compared the changes in urinary hydration markers in a fresh spot urine sample, no evidence exists pertaining to the proper storage of a 24-hour urine sample, with the latter being the current clinical standard method of assessing day-to-day hydration status [2,3,13–15]. Furthermore, prior literature [10–12] has not examined the influence of how the seal of the specimen container, which may alter the rate of sample evaporation, influences 24 h urinary hydration. By minimizing the rate of evaporation, the integrity of urinary hydration markers may be maintained. Thus, the purpose of this study was to examine the effect of storage temperature, duration, and storage vessel seal on 24 h urinary hydration markers. It was hypothesized that refrigerated samples that were double sealed would preserve the integrity of 24 h urinary hydration markers to a better extent than samples stored at room temperature and samples that were stored in containers with only a single seal.

## Materials and methods

### Participants

Twenty-one males (n = 8) and females (n = 13) (mean±SD; age, 24±5 y; body mass, 68.9±24.2 kg; height, 160.2±32.1 cm) without a history of renal disease or currently taking any medications or supplements known to affect the accuracy of urinary hydration markers [16,17] were enrolled in this study. All participants provided written and informed consent for the study, which was approved by the Institutional Review Board at the University of North Carolina at Greensboro and performed in accordance with the World Medical Association’s Declaration of Helsinki.

### Procedures

Participants arrived at the laboratory between the hours of 0600–0900 and were provided a container in which they were instructed to collect their urine output for the next 24 h. Participants returned the following morning ±1 h from the day prior with their urine sample. All urine samples were analyzed within two hours to obtain a baseline measure of 24 h urinary hydration status and then stored in 4 transparent specimen containers (90mL Samco Wide-Mouth Bio-Tite, Thermo Fisher Scientific Inc., Waltham, MA), two of each in the following temperatures: 20°C (Room Temperature) and 7°C (Refridgerated). Additionally, for each temperature condition, one of the two vessels was sealed only with the manufacturer provided cap (single seal, SS) while the second vessel was sealed with a secondary seal (double seal, DS) (Parafilm M Laboratory Wrapping Film, Bemis Company Inc., Oshkosh, WI) to minimize the extent of moisture loss due to evaporation. All samples were analyzed after 1 (24 h), 2 (48 h), 3 (72 h), 7 (168 h) and 10 (240 h) days following vortexing, which is consistent with prior literature [11]. Rates of evaporation (ml/d and % of sample) of the sample were assessed by weighing the sample to the nearest 0.0001 g (Ranger 3000, OHAUS Corporation, Parsippany, NJ) prior to and following each sampling and measurement timepoint.

All samples were analyzed for urine osmolality (U_OSMO_), urine specific gravity (U_SG_) and urine color (U_COL_). Prior to analyzing each sample, the sample was vortexed for 5 s. U_OSMO_ was determined via freezing point depression (3320 osmometer, Advanced Instruments, Inc., Norwood, MA; reliability, standard deviation <2 mOsm•kg^-1^ H_2_O between ranges 0–400 mOsm•kg^-1^ and <2% mOsm•kg^-1^ H_2_O between ranges 400–2,000 mOsm•kg^-1^) in triplicate or in quintuple if the osmolality readings of the first three measures differed by >3 mOsm•kg^-1^. U_SG_ was measured using an optical refractometer (ATAGO, Tokyo Japan) that was accurate to the ±0.0010 AU and had a resolution of 0.0001. U_COL_ was measured by comparing each urine sample to an 8-point urine color chart [18] where the sample was held against a white background in a well-lit room The same technician held the urine up to the urine color chart at eye level in the same room with adequate lighting.

### Statistical analysis

Statistical analysis was performed using JMP Pro (version 12.1.0., SAS Inc., Cary, NC, USA). To examine the correlation between the baseline sample and the stored samples, regression analyses were utilized, and measures were plotted against the line of identity to show over-under estimation by treatment. Fresh sample versus stored sample values of each storage condition and corresponding time point were evaluated by Bland-Altman analysis [19] comparison for U_OSMO_, U_SG_ and U_COL_. To examine differences between SS and DS U_OSMO_ samples at each time point and temperature, multifactorial ANOVA analysis (condition x time x temperature) was used. Percent difference between each time point (Day 1, 2, 3, 7 and 10) and baseline measures of U_OSMO_ were also calculated. To examine the percent evaporation of the 24 h urine sample across time between samples, a condition x time repeated measures ANOVA with Tukey post hoc analysis was performed. Significance level set a-priori p<0.05 was used.

## Results

**Fig 1:**
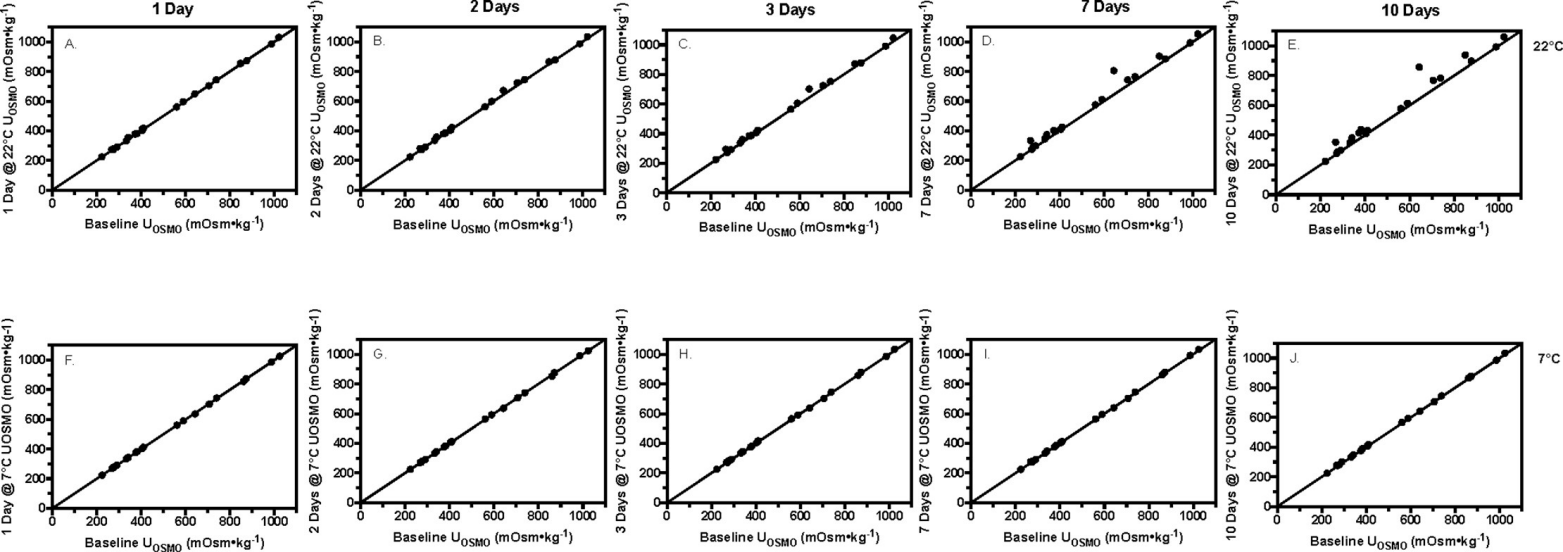
Single sealed stored versus baseline urine osmolality. A-E–values of urine osmolality (U_OSMO_, mOsm•kg^-1^) for the single sealed room temperature (22°C) samples plotted against baseline measures at time points 1, 2, 3, 7, and 10 days. F-J–Values of U_OSMO_ for the single sealed refrigerated (7°C) samples plotted against baseline measures at time points 1, 2, 3, 7 and 10 days.

**Fig 2:**
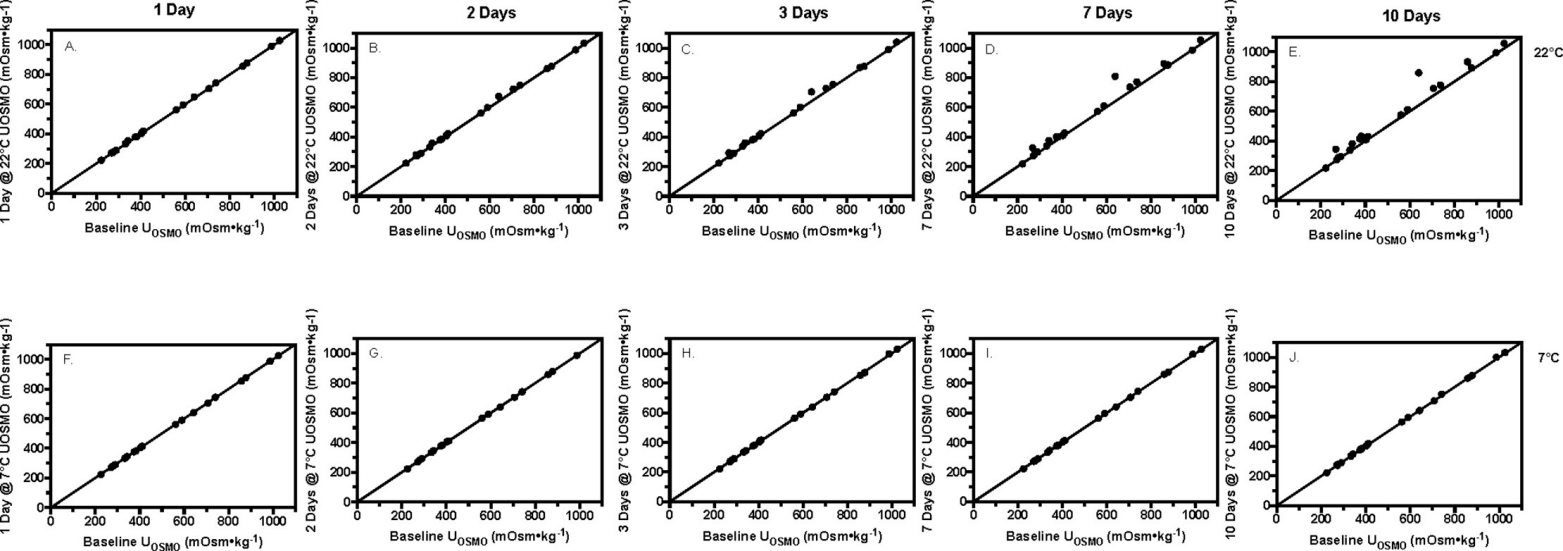
Double sealed stored versus baseline urine osmolality. A-E–values of urine osmolality (U_OSMO_, mOsm•kg^-1^) for the double sealed room temperature (22°C) samples plotted against baseline measures at time points 1, 2, 3, 7, and 10 days. F-J–Values of U_OSMO_ for the double sealed refrigerated (7°C) samples plotted against baseline measures at time points 1, 2, 3, 7 and 10 days.

Bland-Altman analysis for U_OSMO_ is shown in Tables 1 and 2. Storing the samples at 20°C significantly elevated U_OSMO_ measures beginning 1 day after baseline measures were assessed, independent of the method of sealing the specimen container (p<0.002). For refrigerated samples, U_OSMO_ measures for samples stored in SS containers became significantly elevated 3 days following baseline measures (p<0.003), whereas U_OSMO_ measures for samples stored in DS containers became significantly elevated 7 days following baseline measures (p<0.02). Figs 1 and 2 depicts orthogonal diagrams between baseline U_OSMO_ measures and stored samples at 20°C (A-E) and 7°C (F-J) for SS (Fig 1) and DS (Fig 2) specimen containers.

**Table 1:** Bland-Altman Results: Mean Difference in Urine Osmolality of Fresh Sample vs. Single Seal (SS). Upper and Lower 95% CI, and *p*-value.

Storing Temperature	Time (days)	Mean Difference from Fresh (mmol/kg)	Lower 95%	Upper 95%	*p*
	1	+4	+2	+8	**<0.01**
	2	+7	+4	+11	**<0.01**
22°C	3	+12	+6	+19	**<0.01**
	7	+27	+11	+43	**<0.01**
	10	+39	+10	+61	**<0.01**
	1	+1	0	+2	0.19
	2	+1	0	+3	0.06
7°C	3	+3	+1	+4	**<0.01**
	7	+3	+2	+5	**<0.01**
	10	+5	+3	+7	**<0.01**

**Table 2:** Bland-Altman Results: Mean Difference in Urine Osmolality of Fresh Sample vs. Double Seal (DS). Upper and Lower 95% CI, and *p*-value.

Storing Temperature	Time (days)	Mean Difference from Fresh (mmol/kg)	Lower 95%	Upper 95%	*p*
	1	+3	+1	+5	**<0.01**
	2	+7	+3	+11	**<0.01**
22°C	3	+11	+5	+18	**<0.01**
	7	+25	+9	+42	**<0.01**
	10	+36	+14	+57	**<0.01**
	1	0	-1	1	0.9
	2	+1	0	+2	0.06
7°C	3	+1	0	+3	0.2
	7	+2	0	+3	**<0.01**
	10	+3	+1	+5	**<0.01**

Bland-Altman analysis for U_SG_ and U_COL_ is shown in Tables 3 and 4 and Tables 5 and 6, respectively. Conversely to U_OSMO_, U_SG_ measures were significantly elevated in SS and DS samples stored at 7°C beginning 1 day following baseline measures (p<0.005), compared to SS and DS samples stored at 20°C (p>0.05). Out of the samples stored at 7°C, the only sample stored that did not show statistical significance was the SS Day 3 sample (p = 0.88). For U_COL_, measures were significantly elevated beginning at day 7 for SS samples stored at 20°C (p<0.009) and 7°C (p<0.007). For DS samples, U_COL_ measures were significantly elevated at day 7 for samples stored at 20°C (p<0.007) and at day 10 for samples stored at 7°C (p = 0.01).

**Table 3:** Bland-Altman Results: Mean Difference in Urine Specific Gravity of Fresh Sample vs. Single Seal (SS). Upper and Lower 95% CI, and *p*-value.

Storing Temperature	Time (days)	Mean Difference from Fresh	Lower 95%	Upper 95%	*p*
	1	0.000	0.000	+0.001	0.67
	2	0.000	0.000	+0.001	0.38
22°C	3	0.000	0.000	+0.001	0.67
	7	+0.001	0.000	+0.001	0.06
	10	+0.001	0.000	+0.001	0.053
	1	+0.001	+0.001	+0.001	**<0.01**
	2	+0.001	+0.001	+0.001	**<0.01**
7°C	3	+0.000	-0.002	+0.001	0.88
	7	+0.001	0.000	+0.001	**<0.01**
	10	+0.001	0.000	+0.001	**<0.01**

**Table 4:** Bland-Altman Results: Mean Difference in Urine Specific Gravity of Fresh Sample vs. Double Seal (DS). Upper and Lower 95% CI, and *p*-value.

Storing Temperature	Time (days)	Mean Difference from Fresh	Lower 95%	Upper 95%	*p*
	1	0.000	0.000	0.001	0.38
	2	0.000	0.000	0.001	0.13
22°C	3	0.000	0.000	0.001	0.5
	7	0.000	-0.002	0.002	0.33
	10	0.000	0.000	0.001	0.1
	1	+0.001	+0.001	+0.001	**<0.01**
	2	+0.001	+0.001	+0.001	**<0.01**
7°C	3	+0.001	+0.001	+0.001	**<0.01**
	7	+0.001	+0.001	+0.001	**<0.01**
	10	+0.001	0.000	+0.001	**<0.01**

**Table 5:** Bland-Altman Results: Mean Difference in Urine Color of Fresh Sample vs. Single Seal (SS). Upper and Lower 95% CI, and *p*-value.

Storing Temperature	Time (days)	Mean Difference from Fresh	Lower 95%	Upper 95%	*p*
	1	0	-0.2	+0.3	0.67
	2	0	-0.2	+0.1	1.00
22°C	3	+0.2	-0.1	+0.6	0.09
	7	+0.6	+0.2	+1.1	**<0.01**
	10	+1.1	+0.5	+1.74	**<0.01**
	1	+0.1	-0.15	+0.3	0.43
	2	+0.1	-0.15	+0.4	0.43
7°C	3	+0.3	-0.04	+0.6	0.08
	7	+0.7	+0.2	+1.1	**<0.01**
	10	+1.1	+0.5	+1.8	**<0.01**

**Table 6:** Bland-Altman Results: Mean Difference in Urine Color of Fresh Sample vs. Double Seal (DS). Upper and Lower 95% CI, and *p*-value.

Storing Temperature	Time (days)	Mean Difference from Fresh	Lower 95%	Upper 95%	*p*
	1	+0.2	0.0	+0.4	0.1
	2	+0.3	+0.03	+0.6	0.03
22°C	3	+0.2	-0.17	+0.6	0.23
	7	+0.7	+0.2	+1.1	**<0.01**
	10	+0.9	+0.5	+1.4	**<0.01**
	1	0	-0.4	+0.4	1.00
	2	+0.3	0.0	+0.6	0.06
7°C	3	+0.2	-0.4	+0.8	0.53
	7	+0.5	-0.1	+1.03	0.09
	10	+0.8	+0.2	+1.3	**0.01**

**Fig 3:**
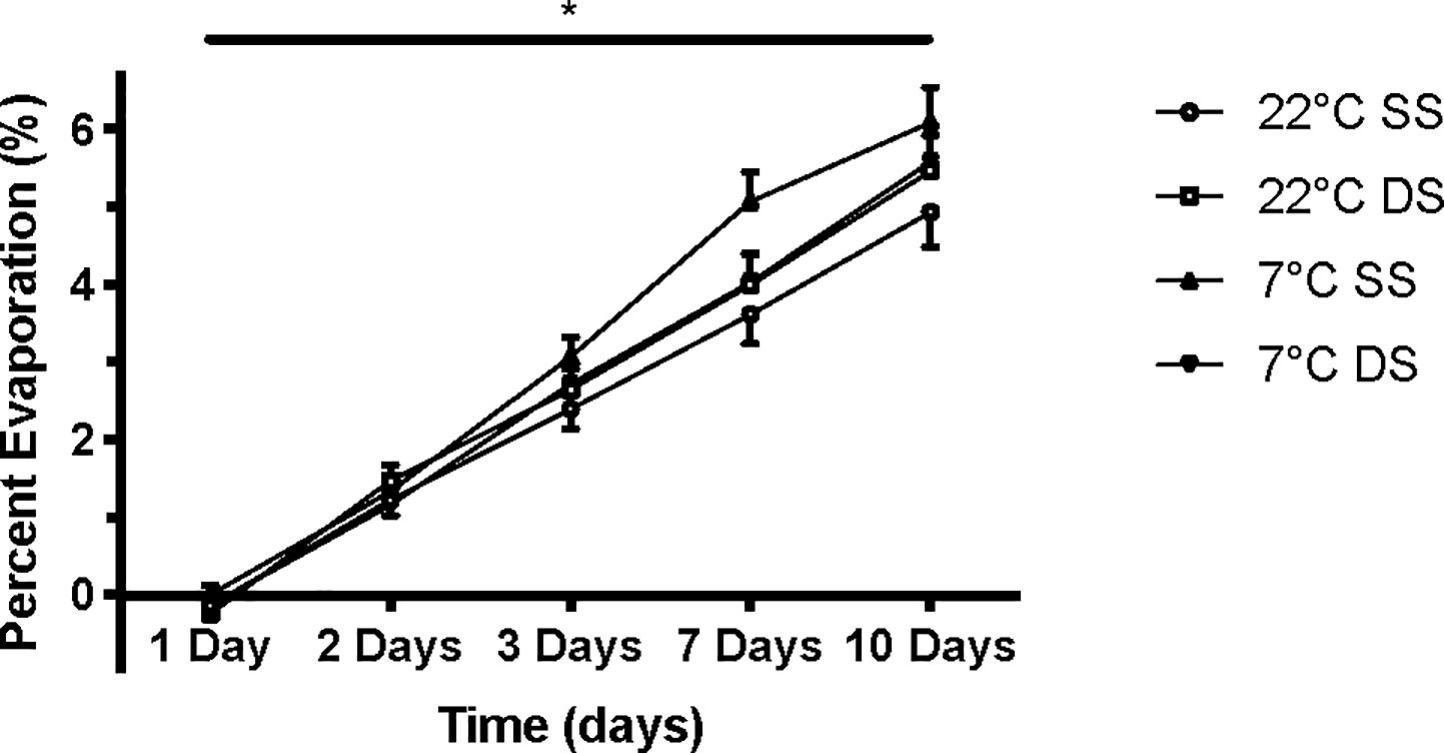
Stored urine sample evaporation. Percent of evaporation (%) at each time point (1 day, 2 days, 3 days, 7 days and 10 days) of samples stored at 22°C and 7°C compared to baseline measures. SS = single sealed sample, DS = double sealed sample.

Multifactorial analysis comparing SS and DS at each temperature and time point revealed that there were no differences in methods by with the specimen containers were sealed (p>0.05). Furthermore, the percent difference in U_OSMO_ between SS and DS measures when compared between each time point and baseline measures, respectively were not significantly different (p>0.05). There was also no influence on storage temperature or method of storage container seal on the percent evaporation of the urine sample (p>0.05); however, there was a significant main effect for time (Fig 3, p<0.05).

## Discussion

The purpose of this study was to determine the efficacy of storage temperature, duration and vessel seal on 24 h urinary hydration markers. We found that the integrity of the 24 h baseline urinary hydration measure was maintained when stored at 7°C for 2 days (48 h) (single-sealed storage container) and 3 days (72 h) (double sealed storage container) for U_OSMO_, a sensitive measure for measuring solute concentration in urine. Our findings also show that U_SG_ measures were maintained when stored at 20°C across 10 days (240 h) and U_COL_ measures were maintained when stored for 3 days (72 h) following baseline measures, regardless of storage temperature or storage container seal. Lastly, we found that the integrity of 24 h urinary hydration markers were not influenced by the sealing method of the storage containers.

The findings from the current study further add to the results from prior literature [10–12] that examined the integrity of urinary hydration measures following defined periods and methods of storage. Unlike previous findings showing U_OSMO_ measures remaining stable when stored at room temperature up to 48 h post baseline measures [10,11], our study found that U_OSMO_ measures were significantly different beginning 24 h post baseline assessment. This could be attributed to our baseline measures were derived from a 24 h sample compared to a spot sample, thus allowing portions of our sample to reside in a room temperature environment for up to 24 h prior to the baseline measure.

Further, and conversely to Adams et al. [11] that found stability in U_OSMO_ measures up to 7 days following baseline, our findings show that U_OSMO_ measures only remain stable when stored in a refrigerated (7°C) environment for 48 h (SS) to 72 h (DS). While our findings found significant differences in U_OSMO_ measures between baseline and following 7 and 10 days of storage, our results showed that the mean difference and 95% confidence intervals for SS (MD [95%CI]; 7 days, 3 mOsm•kg^-1^ [2, 5], p = 0.0003; 10 days, 5 mOsm•kg^-1^ [3, 7], p = 0.0001) and DS (7 days, 2 mOsm•kg^-1^ [0,3], p = 0.0232; 10 days, 3 mOsm•kg^-1^ [1, 5], p = 0.012) samples were small and may not change the interpretation of hydration status in these samples.

Although the examination of storage temperature and duration on U_SG_ and U_COL_ is sparse, our findings support prior findings showing that U_SG_ is unchanged when the sample is stored at room temperature [11,20], even when stored up to 10 days following baseline. Our findings did show, however, that U_SG_ significantly increased following storage in a refrigerated (7°C) environment beginning 24 h following baseline. While this is in contrast to Adams et al. [11] and Hunt et al. [20], one possible explanation for this difference is the temperature of the sample at the time of actual measure. Adams et al. [11] tested their refrigerated samples to once the sample temperature reached room temperature, whereas we tested our samples immediately after retrieving the sample from the 7°C environment. The cool environment may have increased the crystallization of the sediment in our samples, thus altering the specific gravity measure which relies on refraction of light through the medium to derive the measure. U_COL_ measures remained stable in our study for all conditions up to 72 h, which differed from prior literature [11]. The mechanisms for these differences are not clear, however, an initial explanation may be related to the baseline hydration measures. Samples depicting individuals in a well hydrated state (U_COL_ measure < 3 AU) may not undergo many changes due to the ratio of water compared to other solutes in the urine that may alter the sample’s color.

Lastly, this study aimed to examine whether or not the method of sealing the specimen container may have influenced the integrity of the urinary hydration measures. Our rationale is based on the premise that as a liquid evaporates, the concentration of solute within the liquid medium increases, thus altering the resulting hydration measures. We found no significant differences in any urinary hydration marker there were stored in specimen containers that were single or double sealed. This may be due to the fact that the rates of evaporation across conditions were consistent across all samples (Fig 3).

## Conclusion

In conclusion, to achieve optimal results, it is recommended that 24 h urine samples are assessed immediately for the respective hydration markers being analyzed. If immediate analysis is unavailable, such as in the case of field based or longitudinal research, it is recommended that 24 h urine samples are stored in a refrigerated environment and hydration markers (U_OSMO_ and U_COL_) be assessed within 48 h. If assessing U_SG_, caution should be made if assessing the sample immediately following removal from the refrigerated environment as this may alter this hydration marker measure.

## Supporting information

S1 File Urinary hydration marker data. (XLSX)
